# Prevalence and factors associated with smartphone addiction among nursing postgraduates during the COVID-19 pandemic: a multilevel study from China’s mainland

**DOI:** 10.1186/s12888-023-05369-5

**Published:** 2023-12-06

**Authors:** Jie Liu, Xingfeng Yu, Lingna Kong, Xiaobo Zhou

**Affiliations:** 1 Department of Nursing, The First Affiliated Hospital of Chongqing Medical University, Chongqing, China; 2 The Nursing Department, Shaanxi Provincial People’s Hospital, Shanxi, China; 3 School of Nursing, Chongqing Medical University, Chongqing, China; 4 Reproductive Medical Center, The First Affiliated Hospital of Chongqing Medical University, Chongqing, China

**Keywords:** Addiction, Smartphone, Nursing students, Postgraduate, Cross-sectional study

## Abstract

**Background:**

Smartphone addiction is prevalent among college students, and there is a concern that the COVID-19 pandemic may bring an increased prevalence of smartphone addiction due to constant online classes and repeat quarantine policies. This study aims to assess the prevalence and influencing factors of smartphone addiction among Chinese nursing postgraduates during the pandemic by examining variables, including loneliness, perceived stress, resilience, and sense of security.

**Methods:**

This online cross-sectional survey recruited 224 nursing postgraduates in four cities in 2022, using Smartphone Addiction Scale for College Students, the Chinese version of Perceived Stress Scale, UCLA Loneliness Scale Version 3, Chinese version of the 10-item Connor-Davidson Resilience Scale, and the Security Questionnaire. Hierarchical regression analysis and logistic regression analysis were performed to explore the associated factors and predictors of smartphone addiction.

**Results:**

During the COVID-19 pandemic, the prevalence of smartphone addiction was 10.41%. There was a positive correlation between smartphone addiction and loneliness, perceived stress (*P* < 0.001), and a negative relationship with resilience and sense of security (*P* < 0.001). The logistic regression analysis identified five risk factors that contribute to smartphone addiction, including daily duration of using a smartphone (3–5 h) (OR = 11.085, 95%CI = 1.21–101.79), numbers of smartphone (OR = 3.704, 95%CI = 1.33–10.30), perceived stress (OR = 1.163, 95%CI = 1.06–1.28), loneliness (OR = 1.071, 95%CI = 1.01–1.13), age of using a smartphone first time (OR = 0.754, 95%CI = 0.60–0.95). Two protective factors, resilience (OR = 1.098, 95%CI = 1.01–1.20) and sense of security (OR = 0.950, 95%CI = 0.90–1.00), were identified.

**Conclusions:**

Collectively, our study found that during the COVID-19 pandemic, smartphone addiction was prevalent among nursing postgraduates, and loneliness and perceived stress are important risk factors for smartphone addiction. Therefore, administrators should adopt targeted interventions to reduce smartphone addiction and the negative impacts on the psychological well-being of nursing postgraduates during a sudden outbreak of a national epidemic crisis.

**Supplementary Information:**

The online version contains supplementary material available at 10.1186/s12888-023-05369-5.

## Introduction

With more than half of the global population owning a smartphone, problematic smartphone use has become a significant issue worldwide [1, 2]. According to a study published in 2020, about 25.1% of Chinese nursing students suffer from smartphone addiction [3], which is slightly higher than the global prevalence rate of 22% [4]. It has been shown that constant use of smartphones may result in a range of side effects (i.e., negative emotions, changes in lifestyles and behaviors, and lower communication skills) and a flow experience [4–6]. Evidence showed that there was a positive association between flow experience and problematic behavior (i.e., problematic smartphone use) [7–10], and flow experience could mediate the relationship between problematic smartphone use and satisfaction with life among college students [5].

Smartphone usage could be divided into distinct patterns by latent class analysis, and this classification is potentially helpful for identifying risk factors and developing intervention and prevention strategies [11, 12]. Concurrently, some related factors of smartphone addiction were identified, such as demographic characteristics (i.e., gender, women presenting significantly higher scores than men) [4], the distortion of time perception factor having a positive association with problematic gaming behavior [13], and other variables like loneliness and perceived stress [1, 14]. However, the repeat outbreak of COVID-19 pandemic brought various challenges for university students [15]. A range of stressors resulting from COVID-19 (i.e., economic downturn, fear of eruption again and infection, confusion for future and looking for a job, 14-day quarantine) could make them experience physical and mental disorders and even suicidal behaviors [6, 16]. Additionally, previous studies confirmed the frequency and dependence on smartphones have increased during COVID-19 among university students [17] and have exacerbated smartphone addiction [2]. Given this special period, it is meaningful and reasonable to put forward a hypothesis that nursing postgraduates may experience smartphone addiction, but the prevalence and influencing factors remain unknown.

According to Maslow’s hierarchy of needs theory, sense of security is one of the basic needs of people. It is a positive emotion that a person perceives when the environment is safe, stable, peaceful, and free from harm and danger [18]. Prior research posits that sense of security could potentially reduce abnormal behavior like addiction [19]. However, evidence regarding the relationship between smartphone addiction and sense of security is limited [19], especially among Chinese nursing postgraduates. As a global threat and disaster, people with COVID-19 experience psychological disorders. We hypothesized that there is a negative association between sense of security and smartphone addiction among Chinese nursing postgraduates during COVID-19. To test this hypothesis, sense of security was included in our study.

Loneliness is defined as a negative emotion of subjective perception, associated with a poor social system or a lack of close companions [20]. Loneliness is a serious problem among young adolescents, and they may seek various ways to cope with this problem, for example, problematic mobile phone use [21]. However, the relationship between loneliness and smartphone addiction remains elusive. Sonmez et al. found a positive relationship between loneliness and smartphone addiction among nursing students. While a study in 2018 reported no relationship between smartphone addiction and loneliness in high school and university students [14, 22]. To assess the association between the two variables among nursing postgraduates, loneliness was included in our study.

Perceived stress is conceptualized as the perception of stressors and the ability to cope with the threat [23]. Current evidence suggests that stress has a small to medium positive association with smartphone use [24], and it is an influencing factor in smartphone addiction among middle school students [25]. Some studies concluded that stress could cause smartphone use [26], while others proposed that smartphone use may lead to stress [27]. To investigate the relationship between stress and smartphone addiction among Chinese nursing postgraduates during the pandemic, perceived stress was included in our study.

Resilience is characterized by an individual’s ability to adapt to the external environment [28]. As a multidimensional construct, resilience is negatively associated with the pathophysiology of addictive disorders [29], including smartphone addiction. Meanwhile, resilience is also a protective factor against smartphone overdependence [30, 31]. In our study, we tried to explore the effects of resilience on smartphone addiction among Chinese nursing postgraduates during the pandemic.

To address the gap in knowledge regarding smartphone addiction and its influencing factors and predictors among Chinese nursing postgraduates during the COVID-19 pandemic, this study is aimed at (i) understanding the prevalence of smartphone addiction among Chinese nursing postgraduates, (ii) clarifying the relationship between smartphone addiction, loneliness, perceived stress, resilience, and sense of security among nursing postgraduates, and (iii) determining the influencing factors and predictors of smartphone addiction in Chinese nursing postgraduates. We hope that our results will offer valuable insights into preventing and addressing smartphone addiction during a sudden outbreak of a national epidemic crisis.

## Materials and methods

### Participants

A total of 224 nursing postgraduates were enrolled in this study, of whom 221 completed the questionnaire with a high recovery rate of 98.66%. The inclusions were: (i) being a full-time nursing postgraduate, including Ph.D. students; (ii) being a registered student, (iii) having the ability to comprehend the questionnaire, (iv) volunteering to participate in this survey. The exclusions were: (i) nursing postgraduates who had already completed their master's or doctorate; and (ii) those who declined to participate in the study.

### Data collection

To recruit participants for this cross-sectional study, we used the convenience sampling and snowball sampling methods to reach out to nursing postgraduate students from five universities located in different regions of China (two in the northwest, two in the southwest, and one in the east) between April and October of 2022. Due to the COVID-19 pandemic, we employed an online platform called wenjuanxing to distribute the questionnaires. This application (APP) was a universal and stable tool for editing and sending questionnaires in China. The authors shared the links of questionnaire and associated tips with potential respondents one by one, and the other researchers, and the latter, kindly shared the link with their contacts. In total, 224 nursing postgraduates completed the questionnaire via this online tool.

### Measures

The current study used a three-part questionnaire for data collection. The first part examined demographic characteristics (Table 1), while the second part focused on smartphone-related information such as numbers of smartphone, age of using a smartphone first time, daily duration of using a smartphone, and daily frequency of using a smartphone (Table 1). The third part consisted of scales used to assess different constructs.

**Table 1:**
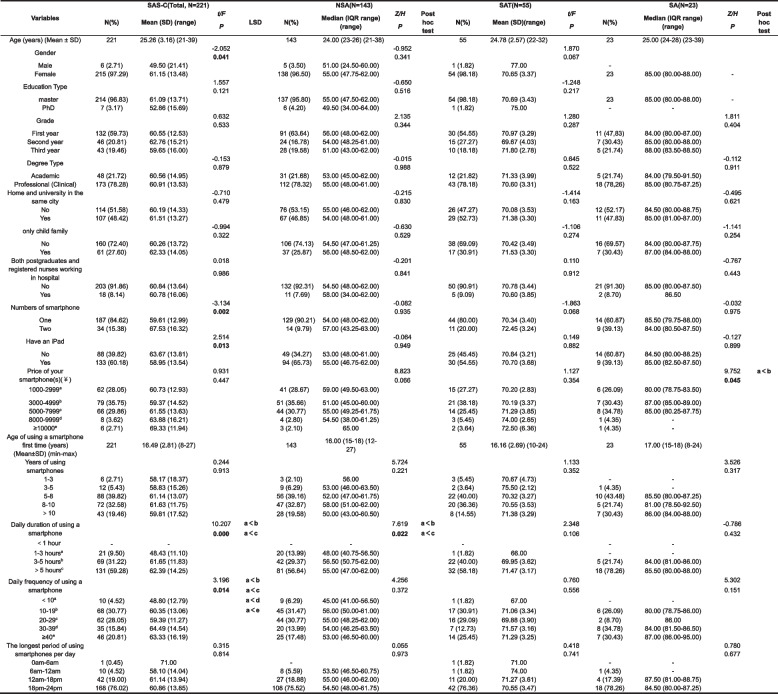
Demographic characteristics and univariate analyses of the factors with SAS-C scores ofnursing postgraduates (*N* = 221)

*IQR* Interquartile Range, *SAS-C* The Smartphone Addiction Scale for College Students

Considering the cultural adaptability and study setting, we chose the Smartphone Addiction Scale for College Students (SAS-C) for this research. A new instrument developed by Su et al. [32] was used to measure the level of smartphone addiction and had six factors with 22 items: withdrawal behavior (7 items), salience behavior (3 items), social comfort (3 items), negative effects (4 items), use of application (App) (3 items), and renewal of App (2 items). The total Cronbach’s α reliability value was 0.88, and 0.81, 0.71, 0.50, 0.85, 0.44, 0.68 for six factors, respectively, and the total test–retest reliability coefficient was 0.93, and 0.82, 0.72, 0.77, 0.74, 0.73, 0.74 for six factors, respectively. Participants responded on a 5-point scale ranging from 1 (not at all true) to 5 (always true). The total score of smartphone addiction in the SAS-C ranged from 22 to 110, and a higher score indicates a higher level of smartphone addiction. According to previous research, above 77 points were regarded as the smartphone addiction group, 66–77 points were considered the smartphone addiction tendency group, and below 66 points indicate the non-smartphone addiction group [33, 34]. Here, the total Cronbach’s α was 0.897, and 0.839, 0.741, 0.627, 0.838, 0.583, 0.681 for six factors, respectively.

The University of California at Los Angeles Loneliness Scale Version 3 (UCLA-LS-V3) comprises 20 items, with 9 items receiving reverse scores, which were used on a 4-point Likert scale from 1 (never) to 4 (always). The total score varied from 20 to 80, with scores of 2 SDs above the mean indicating a very high level of loneliness, whereas a score that is 1 SD above the mean indicates a moderately high level of loneliness, based on Russell’s suggestion. The internal consistency coefficient α ranges from 0.89 to 0.94. In the present study, Cronbach’s α was 0.895.

The Chinese version of Perceived Stress Scale (CPSS) was widely used to examine the level of stress students perceived. It included 14 items, with Cronbach’s α being 0.83, and had been adopted by rural Chinese men with good reliability and validity. The scale had Likert-5 scores from 0 (never) to 4 (very often), with an overall score of 0 to 64. A higher score means perceived stress is severe, and vice versa. In this study, Cronbach’s α was 0.808.

The Chinese version of the 10-item Connor-Davidson Resilience Scale (CD-RISC-10) is a single-dimension scale with 10 items. The scale was formed by several professionals through a two-stage process of translation and back translation, cultural adaptation, validation, and application, which has good psychometric properties. Participants were asked to respond on a 5-point Likert scale which scored from 0 (never) to 4 (almost always). The total score of the scale is the sum of each item (0–40), with a cut-off of 25.5. In this study, Cronbach’s α coefficient was 0.932.

The Security Questionnaire (SQ) has good reliability and validity, with a total Cronbach’s α 0.796, and for the two dimensions, 0.747 and 0.720, respectively. It has been widely used by various Chinese populations. The questionnaire includes two dimensions: interpersonal security and certainty in control. Interpersonal security was mainly used to reflect the individual's safety experience in interpersonal communication, and certainty of control is often employed to assess the individual’s expectations of life. Each dimension has 8 items, for a total of 16 items. Participants were asked to answer a 5-point Likert scale, ranging from 1 (always true) to 5 (not at all true), with total scores from 16 to 80. A higher score means a higher level of security. In the current study, the total Cronbach’s α was 0.918, and 0.858, 0.850 for each dimension, respectively.

### Statistical analysis

Data analysis was performed using SPSS 26.0. Missing values and outliers were examined prior to analysis. Then, the distribution of continuous variables was measured by Kolmogorov–Smirnov test, Shapiro–Wilk test, skewness, kurtosis values, P-P Plot, and scatterplot (Fig. S1 and Fig. S2). Loneliness, perceived stress, and resilience were found to not be normally distributed. Mean, standard deviation, percentages, and numbers were applied to test demographic information, smartphone-related information, and smartphone addiction and sense of security. The median and interquartile range (IQR) were conducted to present loneliness, perceived stress, and resilience. Student’s *t*-test, one-way ANOVA, Mann–Whitney U test, and Krukal-Wallis-H were used to compare the difference between the demographic information among students and sub-groups of smartphone addiction. A one-way ANOVA (and nonparametric or mixed) was applied to compare the difference in loneliness, perceived stress, resilience, and sense of security between three groups of smartphone addiction. To determine the correlation between smartphone addiction, loneliness, perceived stress, resilience, and sense of security. A Spearman correlation analysis was performed. Finally, hierarchical regression analysis was implemented to assess the influencing factors of smartphone addiction, including variables significantly associated with scores of smartphone addiction in the univariate analysis (*P* < 0.05 level) as well as loneliness, sense of security, perceived stress, and resilience. Lastly, the binary multivariable logistic regression model was employed to examine the predictors for the level of smartphone addiction. A two-tailed *P* value less than 0.05 was accepted as a significant difference.

## Results

### Demographic characteristics

A total of 224 participants answered the online survey, of which 221 responses were valid (97.29% females and 2.71% males). The participants had an average age of 25.26 ± 3.16 (ranging from 21 to 39), and the majority of the participants (96.84%) were pursuing a master’s degree. 59.73% were in their first year, and 78.28% were enrolled in a professional (clinical) degree program. More than half of them attended universities in the southwest, but their homes were in different cities. 72.40% reported having a brother or sister, and a small proportion (8.14%) were registered nurses working in a hospital.

### Smartphone-related information

All 221 participants owned a smartphone or laptop. Almost one-fifth (15.38%) owned two smartphones, while 39.82% did not own an iPad. The majority (65.61%) purchased their smartphones for a price range of $3000 to $7999. The average age of first-time smartphone users was 16.49 ± 2.80 (ranging from 8 to 27), and 39.82% of the nursing postgraduates reported using smartphones for 5–8 years. More than half of them (59.28%) spent more than 5 h on smartphones every day, and over half of them used smartphones 10–29 times every day. The period between 6 p.m. and midnight was the most popular time for smartphone use (76.02%). During the COVID-19 pandemic, the participants preferred to use social and entertainment apps rather than study-related apps. The top three most popular apps were WeChat, TaoBao, and Tencent QQ.

### Descriptive statistics and correlation coefficient matrix

**Fig. 1:**
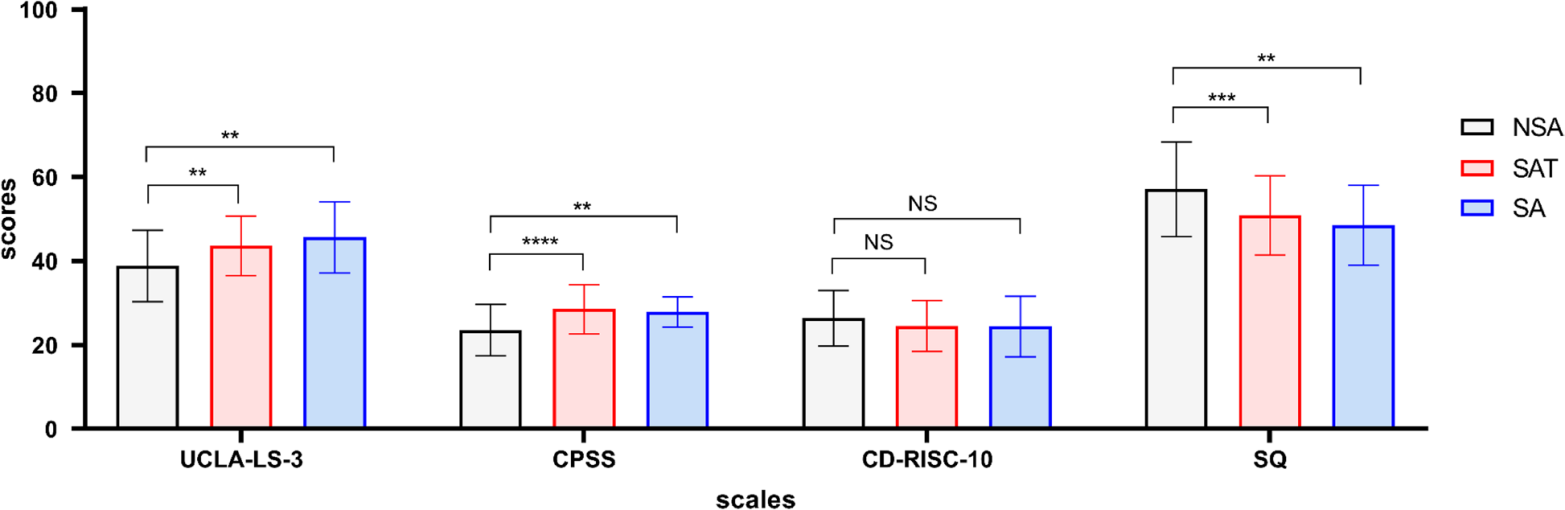
One-way analysis and Kruskal–Wallis test of UCLA-LS-V3, SQ, CPSS, CD-RISC-10 in sub-groups of SAS-C; SAS-C: the Smartphone Addiction Scale for College Students;UCLA-LS-V3, the University of California at Los Angeles Loneliness Scale Version 3; CPSS, the Chinese version of Perceived Stress Scale-10; CD-RISC-10, the Chinese version of the 10-item Connor-Davidson Resilience Scale; SQ, the Security Questionnaire; NSA: non-smartphone addiction; SAT: smartphone addiction tendency; SA: smartphone addiction. **P < 0.01,***P < 0.001, ****P < 0.0001

Tables 2 and 3 explain means, standard deviations, medians, interquartile ranges, and correlation coefficient matrices for smartphone addiction, loneliness, perceived stress, resilience, and sense of security. The scores were 60.83 ± 13.81, 42.00 ± 14.00, 26.00 ± 7.00, 26.00 ± 10.00, and 54.63 ± 11.18 for smartphone addiction, loneliness, perceived stress, resilience, and sense of security, respectively. Smartphone addiction was positively correlated with loneliness and perceived stress (*r* = 0.309, *P* < 0.001, *r* = 0.415, *P* < 0.001). Additionally, smartphone addiction was negatively related to resilience and sense of security (*r* = -0.237,* P* < 0.001, *r* = -0.397, *P* < 0.001). Figure 1 shows that the scores of loneliness, perceived stress, and sense of security were significantly different between non-smartphone addiction and smartphone addiction tendency, non-smartphone addiction and smartphone addiction. There was no significant difference between smartphone addiction tendency and smartphone addiction, and there was no significant difference in resilience among the three groups.

**Table 2:**
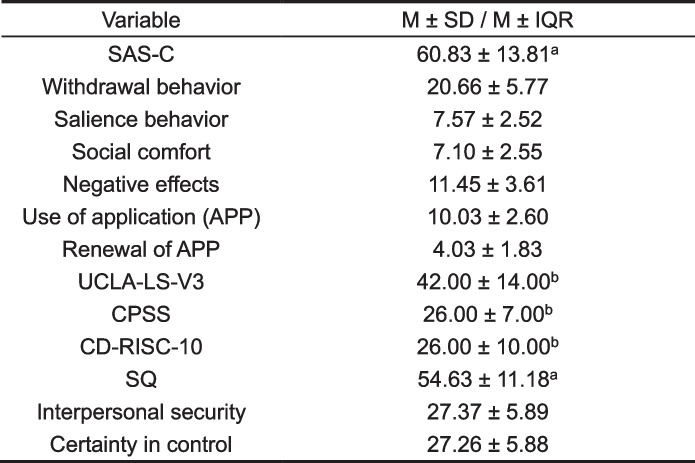
Descriptive statistics of SAS-C, UCLA-LS-V3, CPSS, CD-RISC-10,SQ (*N*=221)

*SAS-C* The Smartphone Addiction Scale for College Students, *UCLA-LS-V3* The University of California at Los AngelesLoneliness Scale Version 3, *CPSS* The Chinese version of Perceived Stress Scale-10, *CD-RISC-10* The Chinese version of the 10-item Connor-Davidson Resilience Scale, SQ The Security Questionnaire

^a^Mean± Standard Deviation

^b^Median± Interquartile Range

**Table 3:** Correlation coefficient matrix of SAS-C, UCLA-LS-V3, CPSS, CD-RISC-10, SQ (*N *= 221)

Variable	1	2	3	4	5	6	7	8	9	10	11	12	13
1. SAS-C	1.000												
2. Withdrawal behavior	0.852^**^	1.000											
3. Salience behavior	0.615^**^	0.384^**^	1.000										
4. Social comfort	0.663^**^	0.441^**^	0.452^**^	1.000									
5. Negative effects	0.710^**^	0.415^**^	0.426^**^	0.438^**^	1.000								
6. Use of application (APP)	0.624^**^	0.524^**^	0.232^**^	0.251^**^	0.410^**^	1.000							
7. Renewal of APP	0.572^**^	0.446^**^	0.300^**^	0.439^**^	0.287^**^	0.184^**^	1.000						
8. UCLA-LS-V3	0.309^**^	0.194^**^	0.234^**^	0.444^**^	0.222^**^	0.090	0.289^**^	1.000					
9. CPSS	0.415^**^	0.310^**^	0.253^**^	0.380^**^	0.373^**^	0.176^**^	0.281^**^	0.440^**^	1.000				
10. CD-RISC-10	-0.237^**^	-0.137^*^	-0.225^**^	-0.369^**^	-0.193^**^	-0.001	-0.240^**^	-0.449^**^	-0.541^**^	1.000			
11. SQ	-0.397^**^	-0.287^**^	-0.288^**^	-0.417^**^	-0.294^**^	-0.179^**^	-0.238^**^	-0.594^**^	-0.513^**^	0.639^**^	1.000		
12. Interpersonal security	-0.379^**^	-0.265^**^	-0.278^**^	-0.401^**^	-0.274^**^	-0.219^**^	-0.206^**^	-0.587^**^	-0.451^**^	0.580^**^	0.944^**^	1.000	
13. Certainty in control	-0.385^**^	-0.287^**^	-0.268^**^	-0.395^**^	-0.292^**^	-0.131	-0.271^**^	-0.536^**^	-0.536^**^	0.633^**^	0.946^**^	0.796^**^	1.000

SAS-C The Smartphone Addiction Scale for College Students, UCLA-LS-V3 The University of California at Los Angeles Loneliness Scale Version 3, CPSS The Chinese version of Perceived Stress Scale-10, CD-RISC-10 The Chinese version of the 10-item Connor-Davidson Resilience Scale, SQ The Security Questionnaire. *P＜0.05, **P＜0.01

### Prevalence and associated factors of smartphone addiction among nursing postgraduates in the univariate analysis and hierarchical regression analysis

The prevalence of smartphone addiction among nursing postgraduates was 10.41% (23/221) in our study. Specifically, the prevalence of smartphone addiction among females was 10.70 (23/215), whereas there was no male student in the smartphone addiction group. Univariate analysis showed that there were significant differences between smartphone addiction and genders (*P* < 0.05), numbers of smartphone (*P* < 0.01), having an iPad (*P* < 0.05), daily duration of using a smartphone (*P* < 0.01), and daily frequency of using a smartphone (*P* < 0.05) (Table 1).

A hierarchical regression analysis was conducted to identify the factors associated with the scores of smartphone addiction (Table 4). The results revealed that gender, numbers of smartphone, daily duration of using a smartphone, daily frequency of using a smartphone, as well as loneliness, perceived stress, and sense of security, were all significantly associated with smartphone addiction. Model 1, which comprised demographic and smartphone-related information, explained 15.3% of the variance in smartphone addiction (*R*^2^ = 0.153, *P* < 0.001). Model 2, which was used to examine the scales as independent variables on smartphone addiction, explained 31.2% of the variance (*R*^2^ = 0.312, *P* < 0.001), and the R^2^ change was 0.164 compared with Model 1.

**Table 4:** Hierarchical regression analysis of SAS-C (*N *= 221)

	Model 1						Model 2					
	Adjusted R^2^: 0.153, R^2^ change: 0.195, F: 4.603, *P*＜0.001						Adjusted R^2^: 0.312, R^2^ change: 0.164, F:7.662,* P*＜0.001					
	B	SE	Beta	t	*P*	95%CI	B	SE	Beta	t	*P*	95%CI
(Constant)	28.458	10.230		2.782	0.006	8.290, 48.626	16.897	13.941		1.212	0.227	-10.590, -44.384
Age	-0.084	0.357	-0.019	-0.234	0.815	-0.789, 0.621	0.192	0.324	0.044	0.591	0.555	-0.448, 0.831
Gender	14.027	5.424	0.165	2.586	0.010	3.334, 24.719	11.563	4.966	0.136	2.329	0.021	1.773, 21.353
Numbers of smartphone	6.450	2.482	0.169	2.599	0.010	1.558, 11.342	5.457	2.244	0.143	2.432	0.016	1.033, 9.881
Have an iPad	-3.588	1.852	-0.127	-1.938	0.054	-7.238, 0.062	-1.676	1.694	-0.060	-0.989	0.324	-5.016, 1.665
Age of using a smartphone first time (years)	-0.325	0.405	-0.066	-0.804	0.423	-1.124, 0.473	-0.568	0.371	-0.116	-1.533	0.127	-1.299, 0.163
Daily duration of using a smartphone												
3-5 hours	11.730	3.219	0.394	3.643	0.000	5.383, 18.076	10.412	2.968	0.350	3.509	0.001	4.561, 16.263
＞5 hours	9.411	3.211	0.335	2.931	0.004	3.082, 15.740	8.790	2.930	0.313	3.000	0.003	3.013, 14.568
Daily frequency of using a smartphone												
10-19	11.544	4.439	0.387	2.600	0.010	2.793, 20.295	7.985	4.062	0.267	1.966	0.051	-0.024, 15.993
20-29	10.451	4.438	0.341	2.355	0.019	1.703, 19.199	7.069	4.053	0.230	1.744	0.083	-0.923, 15.060
30-39	13.818	4.807	0.366	2.874	0.004	4.340, 23.295	10.545	4.362	0.279	2.418	0.016	1.946, 19.145
≥ 40	14.486	4.767	0.427	3.038	0.003	5.087, 23.884	10.109	4.356	0.298	2.321	0.021	1.521, 18.698
UCLA-LS-V3							0.262	0.118	0.162	2.216	0.028	0.029, 0.494
CPSS							0.498	0.168	0.226	2.960	0.003	0.166, 0.830
CD-RICS-10							0.155	0.170	0.074	0.912	0.363	-0.180, 0.489
SQ							-0.219	0.102	-0.178	-2.154	0.032	-0.420, -0.019

SAS-C The Smartphone Addiction Scale for College Students, UCLA-LS-V3 The University of California at Los Angeles Loneliness Scale Version 3, CPSS The Chinese version of Perceived Stress Scale-10, CD-RISC-10 The Chinese version of the 10-item Connor-Davidson Resilience Scale, SQ The Security Questionnaire

### Predictors of smartphone addiction among nursing postgraduates

Table 5 revealed that there were five risk factors associated with smartphone addiction, including the daily duration of using a smartphone (3–5 h) (OR = 11.085, 95%CI = 1.21–101.79), numbers of smartphone (OR = 3.704, 95%CI = 1.33–10.30), perceived stress (OR = 1.163, 95%CI = 1.06–1.28), loneliness (OR = 1.071, 95%CI = 1.01–1.13) and age of using a smartphone first time (OR = 0.754, 95%CI = 0.60–0.95). Additionally, two protective factors were identified, including resilience (OR = 1.098, 95%CI = 1.01–1.20) and sense of security (OR = 0.950, 95%CI = 0.90–1.00) in the logistic regression model. Other variables did not show a significant association in the regression equation.

**Table 5:** Logistic regression analysis of the factors associated with the scores of SAS-C above 66 (*N *= 221)

Variable	Beta coefficient	Standard error	Wald c^2^	OR (95% CI)	*P*
Numbers of smartphone	1.309	0.522	6.295	3.704 (1.33, 10.30)	0.012
Age of using a smartphone first time (years)	-0.283	0.117	5.878	0.754 (0.60, 0.95)	0.015
Daily duration of using a smartphone (3-5h）	2.406	1.131	4.521	11.085 (1.21, 101.79)	0.033
UCLA-LS-V3	0.068	0.028	5.755	1.071 (1.01, 1.13)	0.016
CPSS	0.151	0.047	10.353	1.163 (1.06, 1.28)	0.001
CD-RICS-10	0.093	0.043	4.685	1.098 (1.01, 1.20)	0.030
SQ	-0.051	0.026	3.876	0.950 (0.90, 1.00)	0.049
Constant	-14.214	4.880	8.486	0.000	0.004

SAS-C The Smartphone Addiction Scale for College Students, UCLA-LS-V3 The University of California at Los Angeles Loneliness Scale Version 3, CPSS The Chinese version of Perceived Stress Scale-10, CD-RISC-10 The Chinese version of the 10-item Connor-Davidson Resilience Scale, SQ The Security Questionnaire

## Discussion

Our study focused on the prevalence and influencing factors of smartphone addiction among Chinese nursing postgraduates. To our knowledge, our work is the first study to investigate the situation of smartphone addiction during the COVID-19 pandemic that targeted Chinese nursing postgraduates. The main finding was that smartphone addiction was prevalent among nursing postgraduates during a pandemic, and loneliness and perceived stress were important risk factors for smartphone addiction. These findings would contribute to further understanding of smartphone addiction from the perspective of nursing postgraduates, help provide some practical suggestions for reducing the risk of smartphone addiction, and inspire researchers to do some related studies after the sudden outbreak of a pandemic for deep interpretation and clarification of the mechanism of smartphone addiction.

The prevalence of smartphone addiction (10.41%) in our study was lower than that reported in previous studies targeting undergraduates before the pandemic [35–37]. Different samples and instruments might be responsible for explaining this diversity. According to Kwon’s results [38], the scores of smartphone addiction among students with master's and doctorate were lower than those of high school, college, and university. Besides, since young age was positively correlated with the amount of smartphone use and may be at a higher risk for smartphone addiction [39], it is possible that postgraduates experience a relative lower prevalence of smartphone addiction than undergraduates. Additionally, we used a Chinese original measurement to assess smartphone addiction, and our study was conducted during a special period (COVID-19), which may also in part account for the differences observed. In addition, we showed that the prevalence of smartphone addiction was 10.70% among females, while none of the male students were identified as addicted, which is similar to a recent study by Chen et al. [40], who reported that females may prefer to use smartphones for communication with spouses and friends, leading to more time spent on smartphones than men. However, some other studies with a focus on younger adolescents reported no significant difference between genders [41, 42]. Nursing postgraduates have a certain economic status and more spare time; some of them were registered nurses working in hospitals, so they may be more likely to use smartphones to maintain their relationships. However, although we found a significant difference in smartphone addiction between genders, we considered that it should be explained cautiously and rigorously due to the restricted sample size involved in this study.

Longer periods of smartphone use predicted high levels of smartphone addiction. However, there was almost no specific time duration given before [1, 22, 43]. In our study, we found that spending 3–5 h on smartphones is the strongest risk factor for smartphone addiction, and spending 3 h on smartphones may be an important cut-off for predicting smartphone addiction. This investigation has a potentially positive impact on identifying the risk of smartphone addiction following the implementation of further definite interventions to prevent addiction for policymakers in the educational department. However, future studies are required to examine the reliability of this factor in predicting smartphone addiction in diverse regions with larger samples.

Sense of security was a strong protective factor against smartphone addiction. Whether in times of pandemic or not, the scores of sense of security (54.63), interpersonal security (27.37), and certainty in control (27.26) of nursing postgraduates are higher than those of nursing undergraduates [44, 45], suggesting a possible association between education level and sense of security scores. In the present work, we demonstrate that sense of security is negatively correlated with smartphone addiction among nursing postgraduates. Our study was conducted during a period when the epidemic occurred repeatedly, so some students experienced the blockade and even quarantine. Thus, they had to face and cope with multi-level mental disorders (i.e., fear, depression, anxiety, and stress), which can harm the systems of persons, families, and society, based on the social-ecological theory [46]. If they are unable to manage these difficulties, they may feel insecure and spend more time on smartphones. Further intervention studies will be needed to provide valid strategies to improve the level of sense of security, especially for people in crisis.

Loneliness is one of the strongest predictors of smartphone addiction. Herein, we found that nursing postgraduates with loneliness had a high risk of smartphone addiction, this is consistent with the previous study that identified loneliness as the strongest predictor of smartphone addition [47]. This finding could be explained by the lockdown and quarantine strategies adopted during COVID-19, which reduced interpersonal communication and increased seclusion [48]. Additionally, females were more likely to suffer from loneliness and other negative emotions, which also contributed to smartphone addiction [49]. So, it is suggested that measures to relieve loneliness should be taken, such as providing social and psychological support, organizing discussion groups, and developing social skills training programs for policymakers, especially focused on those who are women or show distress and anxiety [50].

Our study also found that nursing postgraduates with high perceived stress scores were at a higher risk of smartphone addiction. It is identical to previous results [51]. As some of the respondents in our study were not only students but also registered nurses working in hospitals, they might have experienced stressors ranging from research to work or family, and stress from COVID-19 would contribute to a high level of perceived stress. Therefore, initiatives such as providing systemic and routine curriculums and lectures on positive psychology both in schools and hospitals, face-to-face or online mindfulness practice, and psychological detachment are encouraged for nursing postgraduates when faced with various stressors [52, 53].

High levels of resilience play a protective role in smartphone addiction. Although the students in this study exhibited a high level of resilience, the level may vary over time if a new calamity occurs. Therefore, long-term strategies are urgently required to enhance resilience, particularly in adverse life circumstances. Current evidence suggests that maintaining self-care, seeking positive support from family, peers, and society, and receiving professional training could be critical elements for building individual resilience that could favorably alleviate smartphone addiction [28]. Future research should focus on the short- and long-term effects of intervention programs to enhance resilience and prevent smartphone addiction among nursing postgraduates.

### Limitations

The study presented some limitations that should be acknowledged. Firstly, the snowball sampling and convenience sampling techniques used may limit the generalizability of the results to other populations or regions. Also, the main respondents in this study were female, which may influence the generalizability. Secondly, the data collected relied on self-reported questionnaires, which may introduce recall bias. Thirdly, too many items to be answered may affect the reliability of the study. Fourth, the cross-sectional study design could not assess the changes in variables over time during COVID-19 among nursing postgraduates. Future studies could use a larger sample size and random sampling techniques to enhance the generalizability of the results. In addition, using a combination of self-reported data and objective measures such as app usage data may reduce recall bias. Finally, further research could use a longitudinal design to investigate the changes in the level of smartphone addiction over time and to evaluate the effectiveness of intervention programs. Further research in larger populations is needed to validate the findings in the current study.

## Conclusions

Our results indicate that Chinese nursing postgraduates experienced a certain prevalence of smartphone addiction during the COVID-19 pandemic. Smartphone addiction was found to be positively correlated with loneliness and perceived stress and negatively correlated with resilience and sense of security during this special period. Seven predictors were included in the logistic regression model, including five risk factors and two protective factors. Our research provides data from a new perspective on sense of security and finds new predictors of smartphone addiction. To prevent and address smartphone addiction during a sudden outbreak of a national epidemic crisis, positive strategies such as mindfulness, meditation, positive psychology, and resilience-building programs should be implemented. Moreover, it is necessary to conduct more investigations on smartphone addiction after the outbreak.

### Supplementary Information

**Additional file 1:** **Fig. S1.** Normal P–P graph of the standardized residual regression. **Fig. S2.** Dispersion graph of the dependent variable ‘SAS-C’.

## Data Availability

The data that support the findings of this study are not shared due to confidentiality offered to survey participants.

## Abbreviations

SAS-C: The Smartphone Addiction Scale for College Students
CPSS: The Chinese version of Perceived Stress Scale
UCLA-LS-V3: The University of California at Los Angeles Loneliness Scale Version 3
CD-RISC-10: The Chinese version of the 10-item Connor-Davidson Resilience Scale
SQ: The Security Questionnaire
