# Supplementary material for: Prevalence and factors associated with smartphone addiction among nursing postgraduates during the COVID-19 pandemic: a multilevel study from China’s mainland
Source: BMC Psychiatry. 2023 Dec 6;23:915. doi: 10.1186/s12888-023-05369-5 (PMC10699056; doi:10.1186/s12888-023-05369-5)
Supplement: Supplementary file 1 — Additional file 1: Fig. S1. Normal P–P graph of the standardized residual regression. Fig. S2. Dispersion graph of the dependent variable ‘SAS-C’. [file 12888_2023_5369_MOESM1_ESM.pdf]

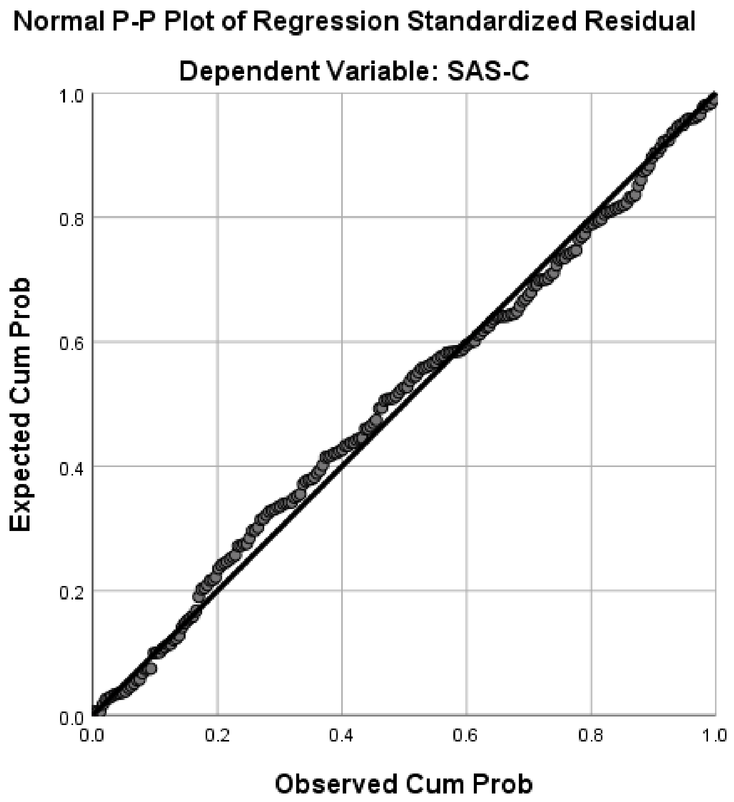

Fig.S1 Normal P–P graph of the standardized residual regression

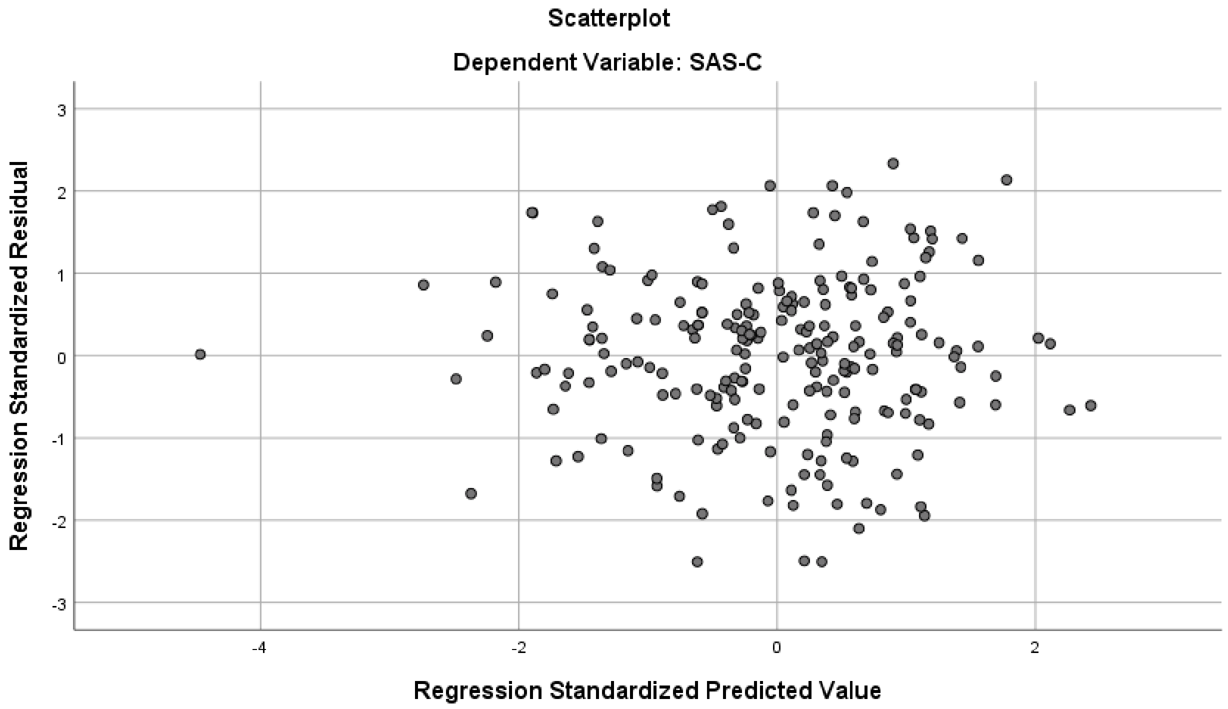

Fig.S2 Dispersion graph of the dependent variable 'SAS-C'
